# The Photocatalytic Activity of CaTiO_3_ Derived from the Microwave-Melting Heating Process of Blast Furnace Slag

**DOI:** 10.3390/nano13081412

**Published:** 2023-04-19

**Authors:** Jun Xie, Qing Ye, Jianghao Zhou, Yue Liao, Gongming Qian

**Affiliations:** 1 School of Resource and Environmental Engineering, Wuhan University of Science and Technology, Wuhan 430081, China; junxie0mineral@wust.edu.cn (J.X.);; 2 Hubei Key Laboratory for Efficient Utilization and Agglomeration of Metallurgic Mineral Resources, Wuhan 430081, China; 3 School of Materials and Metallurgy, Wuhan University of Science and Technology, Wuhan 430081, China

**Keywords:** blast furnace slag, CaTiO_3_, photocatalytic activity, visible light response, degradation mechanism

## Abstract

The extraction of titanium-bearing components in the form of CaTiO_3_ is an efficient utilization of blast furnace slag. The photocatalytic performance of this obtained CaTiO_3_ (MM-CaTiO_3_) as a catalyst for methylene blue (MB) degradation was evaluated in this study. The analyses indicated that the MM-CaTiO_3_ had a completed structure with a special length–diameter ratio. Furthermore, the oxygen vacancy was easier to generate on a MM-CaTiO_3_(110) plane during the photocatalytic process, contributing to improving photocatalytic activity. Compared with traditional catalysts, MM-CaTiO_3_ has a narrower optical band gap and visible-light responsive performance. The degradation experiments further confirmed that the photocatalytic degradation efficiency of pollutants by using MM-CaTiO_3_ was 3.2 times that of pristine CaTiO_3_ in optimized conditions. Combined with molecular simulation, the degradation mechanism clarified that acridine of MB molecular was stepwise destroyed by using MM-CaTiO_3_ in short times, which is different from demethylation and methylenedioxy ring degradation by using TiO_2_. This study provided a promising routine for using solid waste to obtain catalysts with excellent photocatalytic activity and was found to be in keeping with sustainable environmental development.

## 1. Introduction

Titanium-bearing blast furnace slag is a kind of alkaline solid waste formed in the pyrometallurgy process from titanomagnetite and has almost 100 million tons of total quality in the world [1]. The comprehensive utilization of blast furnace slag has become one key sustainability issue confronting the titanium industry in recent years [2]. Generally, the utilization of blast furnace slag is always based on the extraction titanium, which can be divided into acid leaching [3,4], molten salt decomposition [5,6] and carbonization chlorination [7,8,9]. It is further found that the Ca-bearing components are associated with Ti-bearing minerals in blast furnace slag, leading to the difficulty in separation extraction [10]. As perovskite is easily formed in the pyrometallurgical process, it is worth considering the extraction of titanium-bearing components in the form of CaTiO_3_ from the slag.

It has been reported that CaTiO_3_ is a good industrial catalyst alternative for TiO_2_ in photocatalytic degradation pollutants in the environmental remediation field [11,12]. As is well-known, photocatalytic degradation is an effective method to eliminate organic pollutants [13] and exhibits considerable advantages over other physiochemical techniques such as chemical precipitation, adsorption and coagulation. In detail, it can directly degrade pollutants into small molecules represented by water and carbon dioxide to prevent secondary pollution. In addition, photocatalytic degradation relies on solar energy to avoid the environmental problems caused by fossil fuels. Actually, the photocatalytic efficiency depends on the catalytic activity of the photocatalyst [14]. Various chemical methods have been developed to synthesize CaTiO_3_ photocatalysts, including the solid-state reaction [15,16], sol–gel process [17,18], hydrothermal process [19,20,21], etc. Furthermore, precious metal doping [22,23] and organic framework assembly [24,25] are also applied to optimize its catalytic activity. The solid-state synthesis method is based on the solid diffusion reaction at high temperatures. Due to the fast growth rate of an intermediate product, the synthesis of CaTiO_3_ contains impurities with a low specific surface. Solution-phase synthesis methods commonly suffer due to their complex process and are difficult to control, which limits the extensive development. Therefore, the extraction of titanium in the form of CaTiO_3_ from secondary resources to obtain a photocatalyst is an effective solution.

The CaTiO_3_-enriched products have been successfully collected from titanium-bearing blast furnace slag. The obtained catalyst was shown to have a narrower optical band gap than pristine TiO_2_ [26], Ag-doped TiO_2_ NPs [27], and CuO/TiO_2_ [28], which is conducive to higher catalytic activity. MB is a kind of typical pollutant to explore photocatalysis performance due to its recognized carcinogenicity, wide application, and stability under ultraviolet light. In terms of the optimized degradation conditions, the catalytic experiments proved that its performance of degrading pollutants is 2.2 times that of the pristine TiO_2_. Furthermore, the correlations between the crystal structure and catalytic ability are also investigated. In detail, the CaTiO_3_ particles gather and grow to form a complete crystal structure with a special length–diameter ratio induced by the microwave heating effect. In this study, the photocatalytic activity of the obtained CaTiO_3_ from the microwave-melting heating process of a blast furnace was systematically explored through the degradation of MB. It is found that the special crystal structure of the CaTiO_3_ product has a lower banding gap and electron–hole pair recombination rate. Furthermore, the obtained CaTiO_3_ products show the catalytic response under the visible light band based on characterization ability and experimental performance. In addition, combined with molecular simulation and theoretical calculation, the photocatalytic activity of the obtained CaTiO_3_ was compared with that of pristine CaTiO_3_, and the corresponding mechanism for their difference was further discussed and explicated. The obtained catalyst was also found to achieve faster degradation of MB than the pristine TiO_2_, so the degradation mechanism was compared by in situ IR combined with calculated Gibbs free energy. In conclusion, the catalyst with excellent photocatalytic activity was derived from titanium-bearing blast furnace slag in this study, which provided a new idea for solid-waste resource recycling and environmental governance.

## 2. Materials and Methods

### 2.1. Material Preparation

The blast furnace slag of vanadium titanium magnetite used in the experiment came from Panzhihua City in Sichuan Province, China. And contents of TiO_2_ and SiO_2_ were 20.75 wt.% and 25.36 wt.%, respectively. Based on our study, microwave-melting heating followed by gravity separation has been carried out to extract CaTiO_3_ product from blast furnace slag (melting temperature at 1300 °C for pretreatment and microwave heating temperature at 1200 °C for 20 min). The CaTiO_3_ component was enriched and precipitated at the bottom of the heating process. The obtained CaTiO_3_ products were collected and dried in an oven at 105 °C for 4 h, which was named MM-CaTiO_3_.

### 2.2. Experimental Design of Photocatalysis

**Figure 1:**
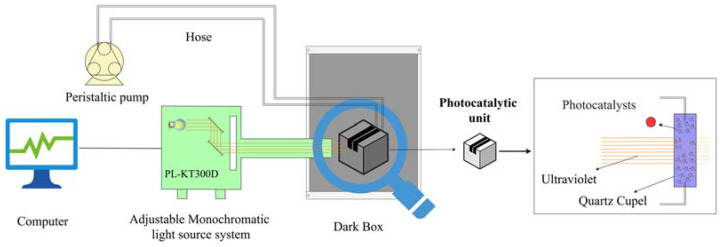
Flow chart of the photocatalytic reaction device.

The photocatalytic reaction was carried out in a circulating reactor equipped with quartz immersion wells, and the photocatalytic reaction device is shown in Figure 1. Experiments were performed using an adjustable monochrome light source system (PL-KT300D, UV region (<390 nm) output power: 2.6 W, Beijing Precise Technology, Beijing, China) to ensure that the photocatalytic process was performed at a known fixed wavelength. The quartz immersion well was experimented with in a camera obscura (Beijing Precise Technology, Beijing, China) to rule out visible light interference. The peristaltic pump (100 rpm, Longer Precision Pump Co. Ltd., Baoding, China) was used to ensure that the photocatalyst powder was in suspension during the experiment. After the photocatalytic degradation experiment, the aqueous mixture was collected and centrifuged to remove the solid catalyst. The MB degradation efficiency (ε) and degradation quantity (Q) were calculated by Equations (1) and (2) [29]. Specifically, the volume of solution used in the experiment is 10 mL; The illumination wavelength was 200 nm to 550 nm, the initial concentration of MB was 2 mg/L to 10 mg/L, and the MM-CaTiO_3_ dosage was 1 g/L to 10 g/L. MB calibration curve formulas such as Equation (3) show that its correlation coefficient (R^2^) is 0.9995 and ranges from 2 mg/L to 10 mg/L,

ε(MB, %) = (C_0_ − C_t_)/C_0_ × 100% (1)

Q(MB, μg) = (Q_0_ − Q_t_) (2)

y = 0.0902x − 0.0094(3)

where C_0_ and C_t_ are initial and residue MB concentrations, respectively; t is time; Q_0_ and Q_t_ are initial and residue MB quantity; y and x represent the absorbance and concentration of MB, respectively.

### 2.3. Instrumental Characterization

X-ray diffraction (XRD, Shimadzu, XD5A, Kyoto, Japan) was used to determine the chemical composition of the catalysts. The morphology and microstructure of catalysts were characterized by scanning electron microscopy (SEM, Fei Company, Nova 400, Hillsboro, OR, USA) and combined with energy dispersive spectroscopy (EDS, Oxford corporation, Penta FET X-3, Abington, UK) analysis to determine the chemical composition. The transmission electron microscope (TEM, JEOL, JEM-2100 UHR STEM/EDS, Kyoto, Japan) was used to determine the orientation of the crystal planes and interplanar spacing of catalysts. The light absorption characteristics and the forbidden band width of catalysts were studied by ultraviolet-visible diffuse reflectance spectroscopy (UV-vis DRS, Shimadzu, UV-2600, Kyoto, Japan). Photoluminescence (PL, Edinburgh Instruments, FLS1000, Edinburgh, UK) spectroscopy with an excitation wavelength of 335 nm was used to study the photoluminescence properties of different catalysts. Photoluminescence quantum yield (PLQY, Edinburgh Instruments, FLS980, Edinburgh, UK) was used to study the utilization efficiency of catalysts for photons. The MB concentration was monitored by measuring the absorbance at 664 nm using the ultraviolet-visible spectrometer (UV-Vis, Shanghai Yidian, Shanghai, China, N4). In situ infrared spectroscopy (In situ IR, Thermo Scientific, Nicolet iS50, Waltham, MA, USA) uses a light wavelength of 300 nm to observe dynamic functional groups in MB solutions. An ion chromatograph (IC, Thermofisher, ICS-6000, Waltham, MA, USA) was used to measure the final product of photocatalytic degradation of methylene blue. GaussView 6.0 was used to calculate the Gibbs free energy of elementary reaction on the degradation process.

### 2.4. First-Principles Theoretical Analysis

The photocatalytic process depends on the reaction in which the catalyst absorbs photons to generate high-energy electrons and holes, then reduction and oxidation reactions are initiated [30]. To reveal the effect of the crystal structure of CaTiO_3_ on the photocatalytic activity, the formation of oxygen vacancy (E_ov_) on different lattice planes was determined using DFT calculation as implemented in the Vienna ab initio Simulation Package (VASP). The E_ov_ values were calculated by Equation (4) [31,32],

E_ov_ = E_ov-1_ − E_pure_ + 1/2 E_O2_ (4)

where E_ov-1_ and E_pure_ represent the gross energy with and without the loss of one oxygen atom, respectively; E_O2_ represents the energy of a single oxygen molecule in the gas phase.

## 3. Results and Discussion

### 3.1. Characterization

**Figure 2:**
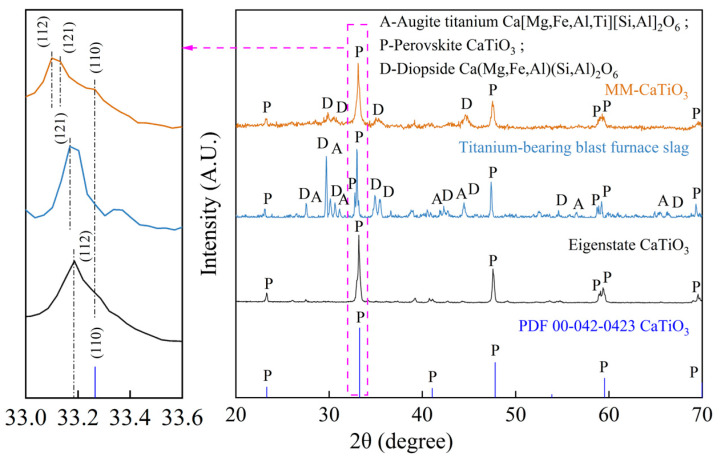
The main phase composition of blast furnace slag and MM-CaTiO_3_.

**Figure 3:**
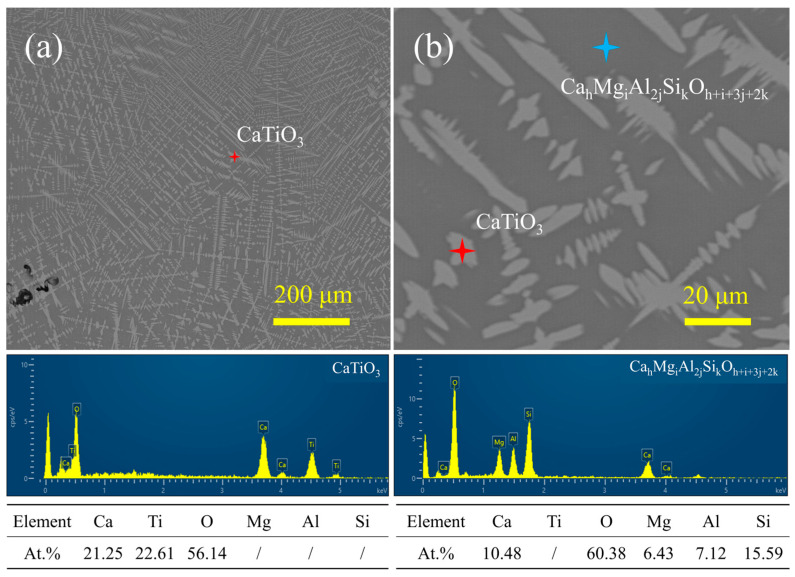
SEM-EDS analysis of MM-CaTiO_3_. (**a**) Mag. 300×; (**b**) mag. 2400×.

Explicating the main phase and morphology structure of MM-CaTiO_3_ is helpful in explaining the difference in their distinguished photocatalytic performance with synthesized CaTiO_3_. The phase composition of blast furnace slag and MM-CaTiO_3_ are shown in Figure 2. The results show that the main Ti-bearing components in the slag are directional transfer to CaTiO_3_ on a microwave-melting heating process. Compared with the predominant orientation of CaTiO_3_ (121) in slag, the MM-CaTiO_3_ has a different plane (112) and a (110) crystal plane. The detailed lattice parameters of CaTiO_3_ are shown in Table 1. The relevant literature reported that the CaTiO_3_ (121) in the space group of Pnma could be transformed into CaTiO_3_ (112) in the space group of Pbnm by rotating 90°, which has the same crystal parameters [33,34]. Hence, CaTiO_3_ (110) is the newly-generated dominant crystal plane in MM-CaTiO_3_. The SEM-EDS analysis of MM-CaTiO_3_ showed that the main component of MM-CaTiO_3_ was CaTiO_3_, as shown in Figure 3a. The micromorphology further indicated that the CaTiO_3_ particle gathers and grows with dense crystal branches and a special length–diameter ratio. In addition, a clear boundary was formed between CaTiO_3_ particles and diopside particles. The results of SEM-EDS analysis with larger magnification (Figure 3b) show that the size of CaTiO_3_ grains is about 5 μm.

**Figure 4:**
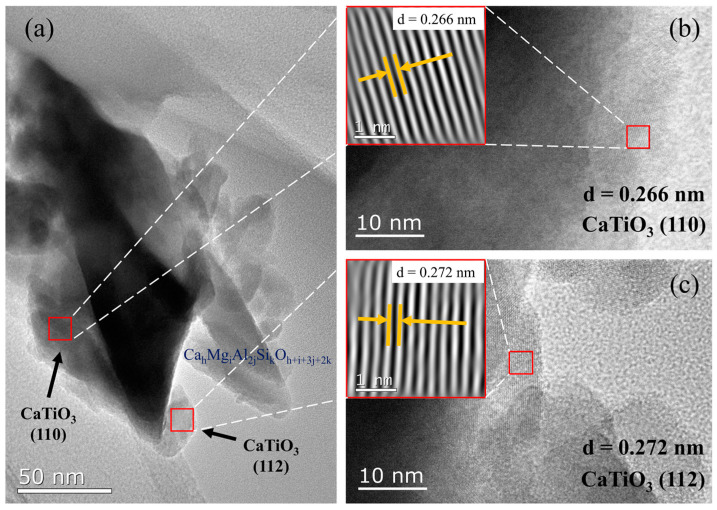
(**a**) TEM image of MM-CaTiO_3_; (**b**,**c**) HRTEM image of CaTiO_3_.

According to the XRD analyses of MM-CaTiO_3_, the interplanar spacing(s) of various CaTiO_3_ crystal orientations were calculated based on the Braggs equation [35,36], as shown in Table 1. Meanwhile, the TEM analyses were used to further determine the newly-generated crystal orientation of MM-CaTiO_3_, as shown in Figure 4. The regional spectrum analyses showed that the main component of the catalyst was CaTiO_3_. The typical oblique downward (110) crystal plane and the almost vertical (112) crystal plane of CaTiO_3_ were founded in the HRTEM image, as shown in Figure 4b,c. The interplanar spacing of (110) and (112) crystal planes were 0.266 nm and 0.272 nm, respectively, which agreed well with the XRD analyses.

**Figure 5:**
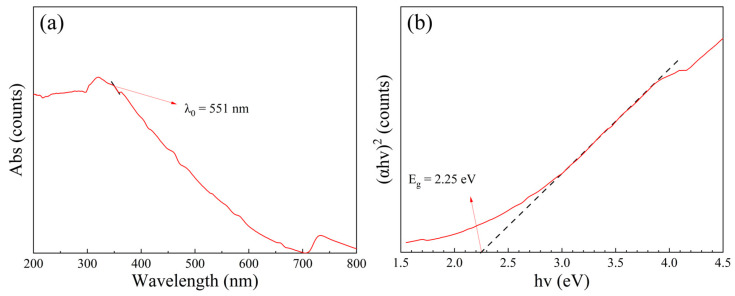
The UV-vis DRS spectrum of MM-CaTiO_3_. (**a**) The threshold wavelength analysis of MM-CaTiO_3_, and (**b**) the forbidden band gap analysis of MM-CaTiO_3_.

**Figure 6:**
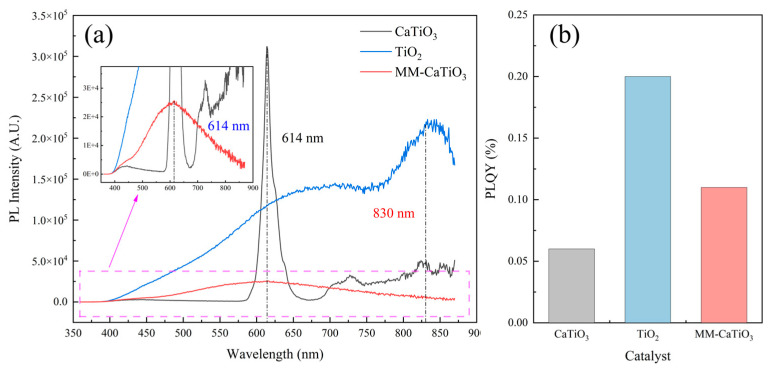
(**a**) The photoluminescence (PL) spectroscopy and (**b**) photoluminescence quantum yield (PLQY) analyses of catalysts.

The photocatalytic property depends on the ability to generate photogenerated electrons and the recombination rate of electron–hole pair, which can be characterized by UV-vis DRS spectrum and PL spectroscopy, as shown in Figure 5 and Figure 6. The UV-vis DRS spectrum analyses show that the threshold wavelength of MM-CaTiO_3_ reaches 551 nm (Figure 5a), indicating that MM-CaTiO_3_ has a wider response frequency band than that of pristine CaTiO_3_ (354 nm) [37] and TiO_2_ (400 nm) [38]. In addition, the threshold wavelength of 551 nm is within the wavelength range of visible light (400 to 760 nm), which indicates that MM-CaTiO_3_ can be used as a visible light-responsive photocatalyst. The band gap (E_g_) is further calculated by the Tauc plot method [39], as shown in Figure 5b. The results show that the E_g_ of MM-CaTiO_3_ is 2.25 eV, which is significantly lower than that of pristine CaTiO_3_ (3.5 eV) [20] and TiO_2_ (3.1 eV) [26]. The relationship between the lattice structure and the band gap is further investigated based on molecular simulation and theoretical calculation. According to the preponderant orientation of MM-CaTiO_3_ and pristine CaTiO_3_, the oxygen vacancy formation energy (E_ov_) on different lattice planes was determined using DFT calculation, as shown in Table 2. The theoretical calculation analyses indicate that the oxygen vacancy is easier to generate on the CaTiO_3_ (110) plane.

Besides the band gap, the recombination of the photogenerated electron–hole pair is also an essential issue impacting the photocatalytic ability of the catalyst. The analyses of photogenerated electron–hole pairs recombination by PL spectroscopy are shown in Figure 6. The MM-CaTiO_3_ and pristine CaTiO_3_ have characteristic peaks at the same wavelength (614 nm), while the characteristic peak wavelength of pristine TiO_2_ at 830 nm. As confirmed in Figure 6a, the MM-CaTiO_3_ has a lower recombination rate of photogenerated electron–hole pairs than both pristine CaTiO_3_ and TiO_2_, which would contribute to its higher photocatalytic activity. Here, the possible mechanism for the recombination rate of the MM-CaTiO_3_ is discussed. As MM-CaTiO_3_ still contains small amounts of diopside, the photogenerated electrons are transferred at the interface of different components that suppress the recombination rate of photogenerated electrons and holes [40]. 

On the other hand, the SiO_2_ tetrahedron structure of the diopside may promote the separation of photogenerated electron–hole pairs [41,42]. The utilization efficiency of photons is further characterized by quantum yield, as shown in Figure 6b. The results show that the quantum yield of MM-CaTiO_3_ is 0.11%, which is much higher than that of pristine CaTiO_3_, indicating a higher photon utilization efficiency, although quantum yields of TiO_2_ reach 0.2%, which is higher than both of MM-CaTiO_3_ and pristine CaTiO_3_. MM-CaTiO_3_ still shows a good catalytic performance considering its lower band gap and recombination rate of photogenerated electron–hole pairs.

### 3.2. Photocatalytic Performance of MM-CaTiO_3_

**Figure 7:**
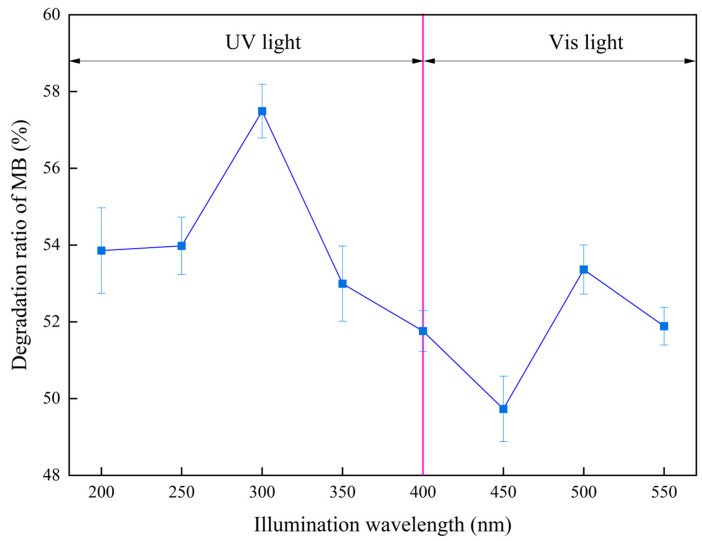
The effect of illumination wavelength on the photocatalysis of MM-CaTiO_3_.

**Figure 8:**
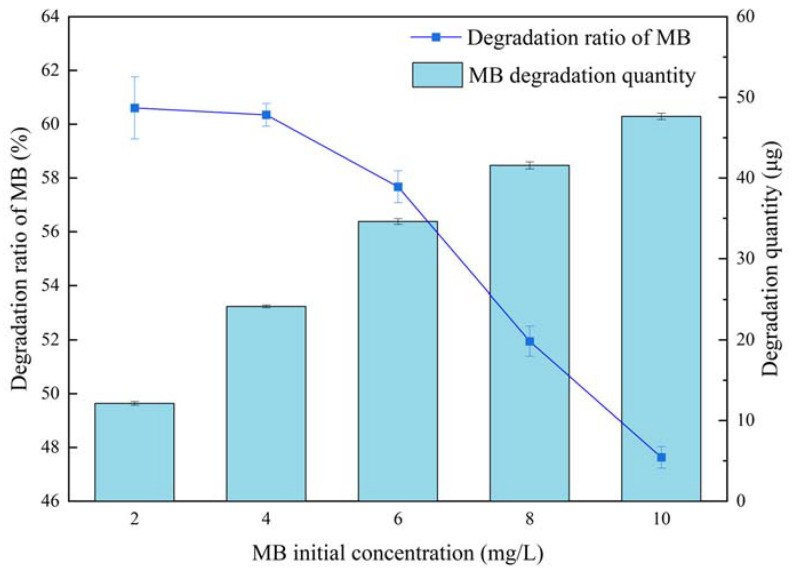
The effect of MB initial concentration on the photocatalysis degradation of MM-CaTiO_3_.

**Figure 9:**
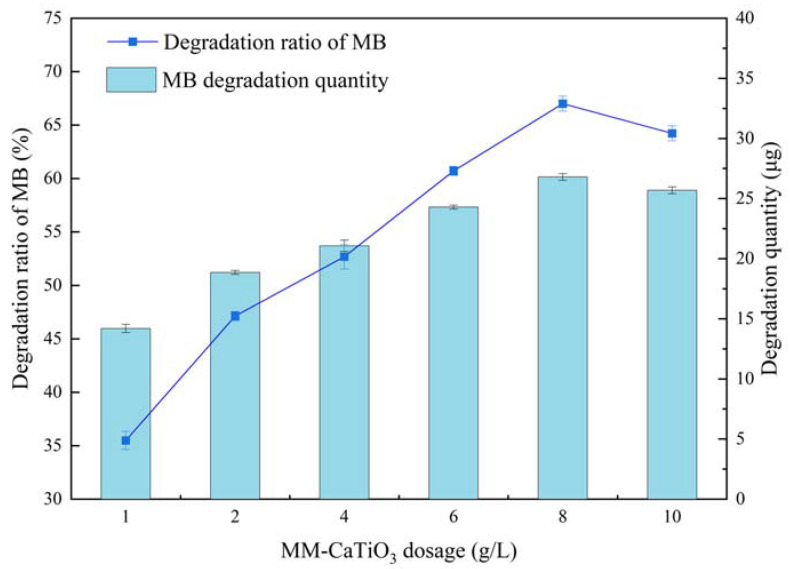
The effect of photocatalyst dosage on the photocatalysis degradation.

The experiments on the photocatalytic degradation of MB by using MM-CaTiO_3_ are systemically investigated, as shown in Figure 7, Figure 8 and Figure 9. The effect of illumination wavelength on the photocatalysis of MM-CaTiO_3_ was investigated on the conditions of initial MB concentration of 6 mg/L and MM-CaTiO_3_ dosage of 6 g/L for illumination time of 1000 s in the light range of 200 nm to 550 nm. Figure 7 shows that the degradation efficiency of MB reached the peak value at 300 nm, which is related to the fixed energy required for photogenerated electrons in MM-CaTiO_3_ to achieve the energy level transition. In addition, it also generated an inflection point at 500 nm in the visible light range [43]. Combined with the previous analysis of photocatalytic property, the newly generated CaTiO_3_ (110) lattice plane was helpful for the response in the visible light range to achieve partial MB degradation, agreed well with the threshold wavelength analysis of MM-CaTiO_3_ (λ = 551 nm) in Figure 5a.

The effect of MB initial concentration was further investigated with a MM-CaTiO_3_ dosage of 6 g/L for an illumination time of 1000 s illumination wavelength of 300 nm. Within the MB initial concentration range of 2 mg/L to 10 mg/L, the degradation efficiency of MB decreased with the increase in the MB initial concentration. The initial excess concentration of MB reduced the transparency of the solution and hindered the UV light absorption of MM-CaTiO_3_, whereas the degradation amount of MB obviously increased as the increasing of MB initial concentration. Based on this, the initial MB concentration of 4 mg/L was appropriate.

The effect of the MM-CaTiO_3_ dosage on photocatalysis was investigated at the illumination wavelength of 300 nm with an initial MB concentration of 4 mg/L for an illumination time of 1000 s. The degradation efficiency of MB gradually increased and then decreased as the MM-CaTiO_3_ dosage increased. The degradation efficiency reached a peak value of 67% with the dosage of 8 g/L. As further increasing the photocatalyst dosage, the degradation efficiency obviously decreased. In addition, the degradation amount of MB showed a similar trend with degradation efficiency. As an excessive dosage of MM-CaTiO_3_ would affect the UV light absorption, the MM-CaTiO_3_ dosage of 8 g/L was suitable for MB photocatalytic degradation.

**Figure 10:**
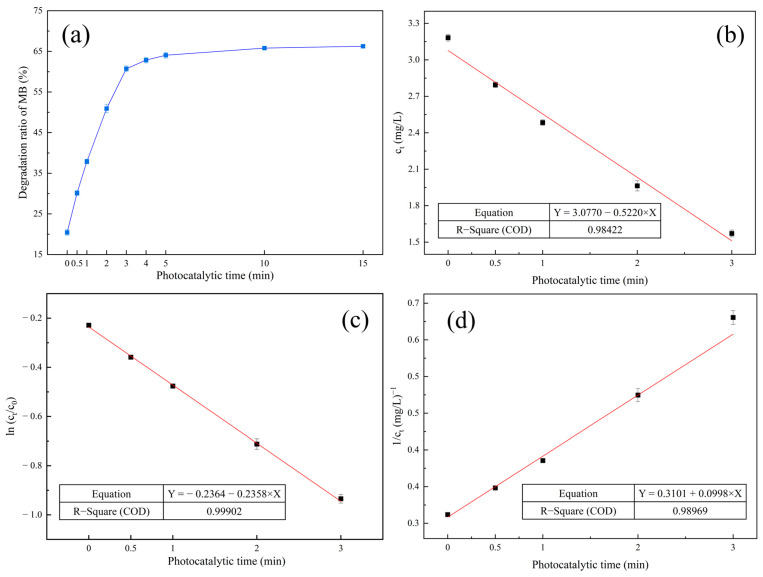
(**a**) The effect of photocatalytic time on the photocatalysis of MM-CaTiO_3_. (**b**) Zero-order reaction kinetics, (**c**) first-order reaction kinetics, and (d) second-order reaction kinetics.

The results of the kinetic study on the photocatalytic degradation of the MB solution are shown in Figure 10 and Table 3. As shown in Figure 10a, the MB degradation efficiency was about 20% when the photocatalytic time was 0. This phenomenon indicated that seldom MB molecular was absorbed on the surface of MM-CaTiO_3_. When the initial MB concentration was 4 mg/L and MM-CaTiO_3_ dosage was 8 g/L, the photocatalytic degradation efficiency of MB increased significantly as the extension of the illumination time. When the photocatalytic time exceeded 3 min, the photocatalytic degradation efficiency of MB increased slowly and then became almost stable at 10 min. In order to clarify the degradation behavior of MB, the typical kinetic models were applied to test the fitness of experimental data. The corresponding equations and parameters are shown in Figure 10. The first-order reaction kinetics of ln(C_t_/C_0_) vs. t resulted in a straight line with higher R^2^ values, which indicated the better applicability of other models. According to the first-order reaction ln(C_t_/C_0_) = −0.2358 t − 0.2364, the rate constant of the photocatalytic degradation reaction was −0.2358 in this study.

**Figure 11:**
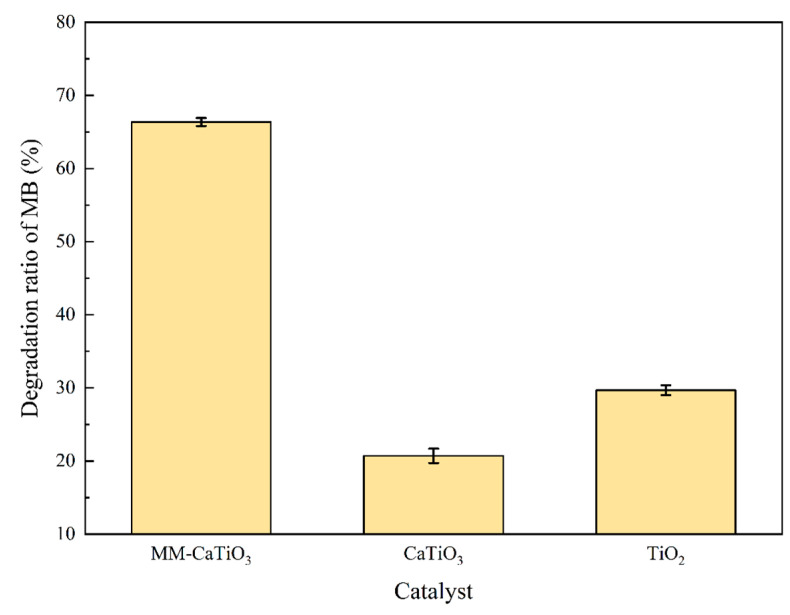
The photocatalytic degradation performance of catalysts.

According to the above analyses of the photocatalytic process, the MB degradation efficiency was further compared under the optimized condition by using different photocatalysts, as shown in Figure 11. The photocatalytic degradation efficiency of MB in the presence of MM-CaTiO_3_ was much higher, which was 3.2 times than that of pristine CaTiO_3_ at an illumination wavelength of 300 nm with an initial MB concentration of 4 mg/L and catalyst dosage of 8 g/L for illumination for 1000 s. The degradation efficiency of MB by pristine TiO_2_ is less than 30%. Based on the crystal structure and photocatalytic property analyses, the MM-CaTiO_3_ with different preponderant lattice orientation contributed to the improved photocatalytic degradation efficiency. In addition, the lower recombination rate of photogenerated electron–hole pairs delays the annihilation of photogenerated electrons and further promotes the degradation of pollutants.

Due to the inconsistency of photocatalytic experimental conditions, especially wavelength and light source power, researchers cannot measure the photocatalytic ability of catalysts by comparing the degradation efficiencies of pollutants. At present, the commonly used measurement method is to compare the band gap of different catalysts. The synthesis process, including raw materials and method of MM-CaTiO_3_, are compared with the previous literature, as shown in Table 4. The results show that the photocatalyst obtained in this study was derived from secondary resources with a narrow band gap by using a simple synthesis process.

### 3.3. Degradation Mechanism of MB Using MM-CaTiO_3_

**Figure 12:**
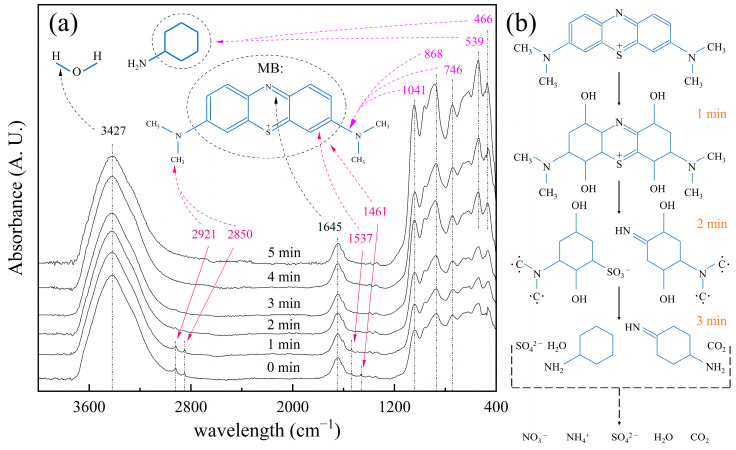
(**a**) In situ IR absorption spectra of MB solution at different time periods; (**b**) flow diagram of photocatalytic degradation of MB by MM-CaTiO_3_.

Furthermore, the mechanism of the photocatalytic degradation of MB using MM-CaTiO_3_ was explored. The in situ IR was used to observe the dynamic behaviors of the functional group on the degradation process, as shown in Figure 12. The MB solution exhibited bands for the C-H asymmetric stretching of CH_3_ at 2921 cm^−1^, the C-H symmetric stretching of CH_3_ at 2850 cm^−1^, the C=N central ring stretching at 1645 cm^−1^, the C=C side ring stretching at 1537 cm^−1^, the acridines stretching at 1461 cm^−1^, the N-C stretching at 1041, 868 and 746 cm^−1^ and the single ring stretching 539 and 466 cm^−1^. The acridines stretching at 1461 cm^−1^ disappeared immediately in the presence of MM-CaTiO_3_ under UV light illumination. As the extension of illumination time, the intensity of C-H asymmetric stretching of CH_3_ at 2921 cm^−1^, the C-H symmetric stretching of CH_3_ at 2850 cm^−1^ and the C=C side ring stretching at 1537 cm^−1^ gradually weakened and then disappeared. However, the N-C stretching at 1041, 868, and 746 cm^−1^ and the single ring stretching at 539 and 466 cm^−1^ were significantly enhanced [46]. This phenomenon indicated that the acridine was directly destroyed by using MM-CaTiO_3_ in a short amount of time. Then, the destroyed acridines further broke the C=C band and demethylated, which then generated small molecules.

**Figure 13:**
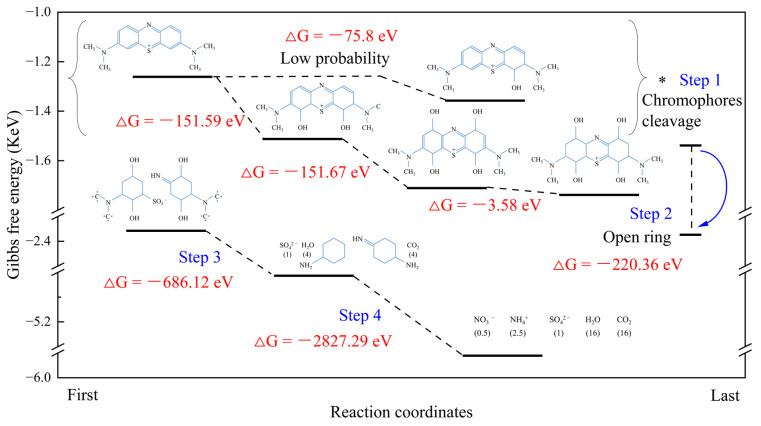
Theoretical calculation and Gibbs free energy of MB-degradation by MM-CaTiO_3_.

The intermediates product and their Gibbs free energies of MB elementary degradation are presented in Table S1 and Figure 13. Obviously, the two hydroxyl radicals preferentially combine with carbon atoms located symmetrically, which has a lower Gibbs free energy reaction than that of a single hydroxyl radical (−75.8 eV). In addition, more hydroxyl radicals react in the symmetry of the benzene ring to promote the further destruction of acridine structure and chromophores cleavage. Furthermore, the enhancement of single-ring stretching foreshadowed the opening ring reactions. Combined with the in situ IR analysis, the demethylation and opening ring reaction almost react at the same time. Finally, the functional group further decomposed to form inorganic ions, H_2_O and CO_2_ [47].

The relevant literature reported that the first step of the demethylation of the MB molecular used TiO_2_. Furthermore, the breaking of the central aromatic ring relied on the transfer of photogenerated electrons and H^+^, thus slowing down the conversion of MB to CO_2_, H_2_O, NH^4+^, and SO_4_^2−^ [48,49]. In contrast, high-activity species were produced in the presence of MM-CaTiO_3_ under UV light illumination, which directly reacted with acridines to achieve stepwise destruction and improve degradation efficiency.

## 4. Conclusions

The titanium-bearing components in the form of CaTiO_3_ derived from blast furnace slag by microwave-melting had excellent photocatalytic properties. This study showed that the MM-CaTiO_3_ obtained a complete crystal structure with a special length–diameter ratio and various predominant crystal orientations. Based on the DFT theoretical calculation, the new-generated CaTiO_3_ (110) plane was easier to form the oxygen vacancy and contributed to the lower band gap than that of pristine CaTiO_3_. The UV-vis DRS spectrum showed that the MM-CaTiO_3_ had a photocatalytic response under visible light, which was further confirmed by the light wavelength experiment of MB degradation. The photocatalytic experiments clarified that the degradation of MB reached 67% at the wavelength of 300 nm with an initial MB concentration of 4 mg/L and dosage of 8 g/L for an illumination time of 1000 s. On the optimized condition, the photocatalytic degradation efficiency by using MM-CaTiO_3_ was 3.2 times that of pristine CaTiO_3_ and 2.2 times that of pristine TiO_2_. The degradation mechanism also indicated that the hydroxyl radical was directly reacted with acridines to achieve stepwise destruction by using MM-CaTiO_3_ in short time, which was different from the gradual decomposition process of demethylation and the MB ring structure breaking by using TiO_2_. Generally, the degradation efficiency of MB using MM-CaTiO_3_ could be further increased by using light with more power. This study provided a promising routine for using solid waste to obtain catalysts with excellent photocatalytic activity and was thereby favorable to sustainable environmental development.

## Figures and Tables

**Table 1:** The lattice parameters of CaTiO_3_ in various materials.

Materials	Titanium-Bearing Blast Furnace Slag	MM-CaTiO_3_		Pristine CaTiO_3_
Space group|number	Pnma|62	Pbnm|62	Pm-3m|221	Pbnm|62
a(Å)	5.4424	5.3796	3.7950	5.3780
b(Å)	7.6417	5.4423	3.7950	5.4440
c(Å)	5.3807	7.6401	3.7950	7.6370
α/β/γ(°)	90.0000	90.0000	90.0000	90.0000
Z	4.00	4.00	1.00	4.00
(h k l)	(121)	(112)	(110)	(112)
d(Å)	2.7025	2.7030	2.6835	2.7030

**Table 2:** The oxygen vacancy formation energy of CaTiO_3_ (112) and (110) lattice plane.

Lattice Plane	(112)	(110)
Molecular model	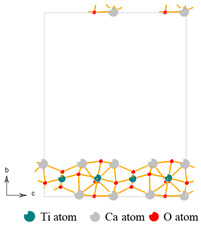	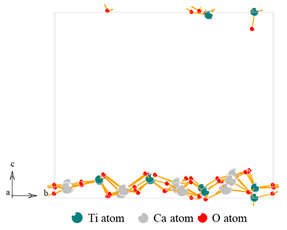
E_pure_	−609.95 eV	−595.48 eV
E_ov-1_	−601.76 eV	−587.59 eV
Oxygen vacancy formation energy	8.152 eV	7.852 eV

**Table 3:** Kinetic reaction model and correlation of photocatalytic degradation.

Model	Equation	R^2^
Zero-order reaction kinetics	C_t_ = −0.5220 t + 3.0770	0.98422
First-order reaction kinetics	ln(C_t_/C_0_) = −0.2358 t − 0.2364	0.99902
Second-order reaction kinetics	1/C_t_ = 0.0998 t + 0.3101	0.98969

**Table 4:** Comparison of the band gap values of photocatalysts in different synthesis processes.

Raw Material	Synthesis Method	Band Gap	Ref.
Calcium carbonate and titanium dioxide.	Calcium carbonate and titanium dioxide are firstly mixed and then calcined at 1400 °C for 2 h.	3.50 eV	[44]
Titanium isoprenoid, calcium acetate and nitric acid.	The Ca^2+^/TiO_2_ sol is first synthesized using calcium acetate, nitric acid and titanium isoprenoid, and then aging and calcined after freeze-drying.	3.44 eV	[18]
Ti(C_4_H_9_O)_4_, AgNO_3_, and CH_3_COOH.	Ag-doped TiO_2_ NPs were synthesized by sol–gel technology.	2.67 eV	[27]
C_12_H_28_O_4_Ti, Cu(NO_3_)_2_, CH_3_COOH, C_3_H_7_OH, and NaBH_4_.	Cu(NO_3_)_2_ is added to the ultrasonically dispersed TiO_2_ NPs aqueous suspension and magnetically stirred. Subsequently, add NaBH_4_ solution and continue stirring for 24 h. Finally, centrifuge, wash, and dry.	2.40 eV	[28]
TiO_2_ (001), TiO_2_ (100), TiO_2_ (101), and Cu(NO_3_)_2_ (0.1 mol/L).	CuO_x_/TiO_2_ catalysts were synthesized by the initial impregnation method using TiO_2_ (001), TiO_2_ (100) and TiO_2_ (101) as the vector and Cu(NO_3_)_2_ (0.1 mol/L) solution as the load.	2.25 eV	[45]
Titanium-bearing blast furnace slag.	Melting pretreatment at 1300 °C for 1 h and then roasted in a microwave field at 1200 °C with a heating time of 20 min.	2.25 eV	This study

## Data Availability

The data presented in this study are available on request from the corresponding author.

## Supplementary Materials

The following are available online at https://www.mdpi.com/article/10.3390/nano13081412/s1, Table S1: The Gibbs free energy of MB molecular and intermediate products.
