# Supplementary material for: The Photocatalytic Activity of CaTiO3 Derived from the Microwave-Melting Heating Process of Blast Furnace Slag
Source: Nanomaterials (Basel). 2023 Apr 19;13(8):1412. doi: 10.3390/nano13081412 (PMC10142369; doi:10.3390/nano13081412)
Supplement: Supplementary file 1 [file nanomaterials-13-01412-s001.zip › nanomaterials-2336350-supplementary.pdf]

Supplementary Materials

The Photocatalytic Activity of $CaTiO_3$ Derived from the Microwave-Melting Heating Process of Blast Furnace Slag

Jun Xie [1], Qing Ye [1,2,*], Jianghao Zhou [3], Yue Liao [1] and Gongming Qian [1,2,*]

[1] School of Resource and Environmental Engineering, Wuhan University of Science and Technology, Wuhan 430081, China; junxie0mineral@wust.edu.cn (J.X.)
[2] Hubei Key Laboratory for Efficient Utilization and Agglomeration of Metallurgic Mineral Resources, Wuhan 430081, China
[3] School of Materials and Metallurgy, Wuhan University of Science and Technology, Wuhan 430081, China
* Correspondence: yeqing@wust.edu.cn (Q.Y.); qiangongming@wust.edu.cn (G.Q.); Tel.: +86-13476834865 (G.Q.)

Table S1. The Gibbs free energy of MB molecular and intermediate products.

Compound	Gibbs free energy/ eV
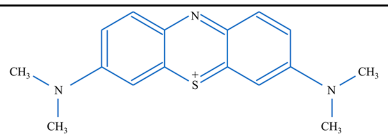	−1182.80
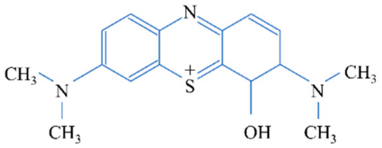	−1258.60
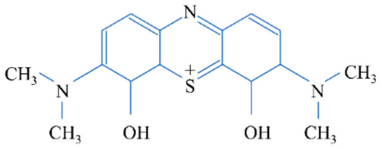	−1334.39
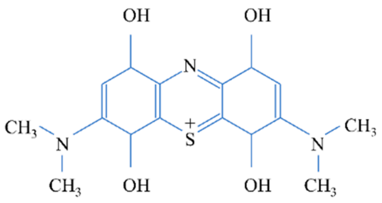	−1486.06
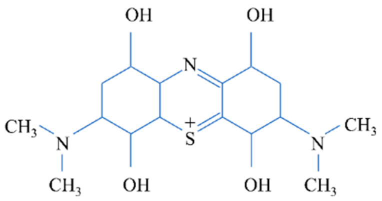	−1489.64
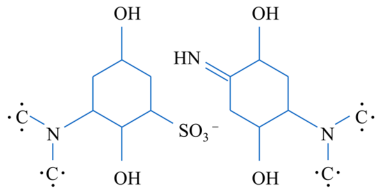	−1710.00

SO_4^{2-} (1) H_2O (4) 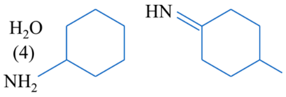 CO_2 (4)	−2396.12
NO_3^- (0.5) NH_4^+ (2.5) SO_4^{2-} (1) H_2O (16) CO_2 (16)	−5223.41
